# Clinical action measures improve the reliability of feedback on quality of care in diabetes centres: a retrospective cohort study

**DOI:** 10.1186/s12913-016-1670-5

**Published:** 2016-08-23

**Authors:** Astrid Lavens, Kris Doggen, Chantal Mathieu, Frank Nobels, Evy Vandemeulebroucke, Michel Vandenbroucke, Ann Verhaegen, Viviane Van Casteren

**Affiliations:** 1 Scientific Institute of Public Health, Rue Juliette Wytsman 14, 1050 Brussels, Belgium; 2 Gasthuisberg KU Leuven, Herestraat 49, 3000 Leuven, Belgium; 3 Onze-Lieve-Vrouwziekenhuis Aalst, Moorselbaan 164, 9300 Aalst, Belgium; 4 AZ Jan Portaels, Gendarmeriestraat 65, 1800 Vilvoorde, Belgium; 5 AZ Sint Maarten, Zwartzustervest 47, 2800 Mechelen, Belgium; 6 ZNA Jan Palfijn, Lange Bremstraat 70, 2170 Merksem, Belgium

**Keywords:** Threshold measure, Clinical action measure, Quality of care, Diabetes, Benchmarking, Feedback

## Abstract

**Background:**

Assessment of quality of care using classical threshold measures (TM) is open to debate. Measures that take into account the clinician’s actions and the longitudinal nature of chronic care are more reliable, although their major limitation is that they require more sophisticated electronic health records. We created a clinical action measure (CAM) for the control of LDL and non-HDL cholesterol from low-complexity data, and investigated how quality of care in individual diabetes centres based on the CAM is related to that based on the classical TM.

**Methods:**

Data was used from 3421 diabetes patients treated in 95 centres, collected in two consecutive retrospective data collections. Patients met the TM when their index value was below target. Patients met the CAM when their index value was below target or above target but for whom treatment initiation or intensification, or possible contraindication, was indicated.

**Results:**

Based on the TM, 60–70 % of the patients received good care. This percentage increased significantly using the CAM (+5 %, *p* < 0.001). At the centre level, the CAM was associated with a higher median score, and a change in position among centres (‘poor’, ‘good’ or ‘excellent’ performer) for 5–10 % of the centres.

**Conclusions:**

Judging quality of diabetes care of a centre based on a TM may be misleading. Low-complexity data available from a quality improvement initiative can be used to construct a more fair and feasible measure of quality of care.

**Electronic supplementary material:**

The online version of this article (doi:10.1186/s12913-016-1670-5) contains supplementary material, which is available to authorized users.

## Background

Since the mid-nineties, several national and international initiatives have been created in order to assess quality of diabetes care [1]. Although they generally use a common set of quality indicators, the purposes of these initiatives differ. Internal quality assessment projects aim to improve quality of care, whereas national efforts rather assess public accountability. Another objective of these initiatives is research where they are used to evaluate programs and assess the effect of policy changes on healthcare quality [2, 3].

Clinical guidelines and indicator selection considerations have led to the development of quality indicators as we know them so far [3, 4]: dichotomous quality indicators covering care processes (such as foot and eye examinations), intermediate outcomes which are immediately influenced by clinical interventions and are predictive for downstream outcomes (such as glycated haemoglobin (HbA1c), low-density lipoprotein (LDL) cholesterol, blood pressure), and end-point outcomes (such as blindness, amputation, cardiovascular complications).

However, cross-sectional threshold measures (TM) based on intermediate outcomes have several limitations and their fairness to assess quality of care is under debate [2, 5–7]. First, there seems to be no single target for all, and the use of more individualized targets based on patient characteristics is increasingly advocated [8, 9]. Secondly, the absolute risk reduction is not a stepwise process, but continuous (and can even be log-linear). Therefore, as a patient approaches the threshold, the marginal benefit of increased treatment decreases while the likelihood of treatment-related side effects and costs increases [2, 6]. Last but not least, all laboratory tests have intrinsic variation [2].

Increasing efforts aim to refine the current quality indicators and develop other types of measures, for instance by extending the TM with patient characteristics and appropriateness of care [2, 6]. One of those new measures is the clinical action measure (CAM). A CAM defines good quality of care not exclusively by a good intermediate outcome, but also by evidence of an appropriate clinical action upon poor control. In addition, by evaluating quality of care within a certain measurement period, it takes into account the longitudinal nature of chronic patient care [10].

The major limitation, however, of constructing more complex quality of care measures such as a CAM is that it requires more structured and sophisticated electronic health records containing explicit data elements reflecting provider actions (such as dates and levels of tests, medication doses) as well as implicit data elements reflecting patient behavior, comorbidity, medication safety and cost concerns [6].

Although these high-complexity data elements are becoming increasingly available, they remain restricted to experimental investigations of new quality indicators and have rarely been applied to large, nationwide initiatives of quality measurement. Therefore, the general framework of this study was to investigate whether more sophisticated indicators, such as a CAM, can be constructed from existing but rather low-complexity data collected cross-sectionally, and how this affects the message to clinicians receiving feedback on the scores.

The specific aims of this study were to investigate (a) whether a CAM can be created for the control of the intermediate LDL and non-high-density lipoprotein (non-HDL) cholesterol using the set of data elements available from a large cross-sectional study; (b) how quality of care defined by the CAM is related to quality of care defined by the classical TM; (c) whether this new measure can be used to give more reliable feedback to the centres in order to improve their quality of care; and (d) whether this principle of measuring quality of care can be generalized for the control of blood pressure.

Data were used from the Initiative for Quality improvement and Epidemiology in patients with Diabetes (IQED), a nationwide quality improvement initiative among insulin-treated diabetic patients attending hospital-based specialist diabetes centres in Belgium [11].

## Methods

### Study population and cohort construction

Data was used from the IQED data collections in 2009 and 2011. The IQED study population is limited to adult (aged ≥18 years) patients with type 1 or type 2 diabetes, treated with at least two insulin injections per day. The IQED data collections took place within a 2 month period. Each centre was asked to complete a standardized electronic questionnaire with the patient’s most recent data by reviewing charts of a 10 % sample of their patients. Patient sampling, based on the last name starting from a random letter, was determined at a national level in the most recent alphabetical patient list until the required number of consecutive patients was reached. The questionnaire contained information on performed processes, intermediate outcomes and complications. The collected data were used to create a nationwide report and provide centres with individual feedback (in which they were compared anonymously to other participating centres, called benchmarking feedback) in order to improve their centre-specific quality of care. The IQED project is managed by our institute. Use of these data has been approved by the Belgian privacy commission (no. 13/092). Details of this quality improvement initiative as well as details of the questionnaire have been published [11] and are available from author by request.

In this study, only patients registered in both 2009 and 2011 were used (*n* = 3808). Data from two consecutive cross-sectional data collections were combined to create one measurement period from 2009 to 2011. The measurement period was defined as the period between the last day of the first of the two consecutive data collections and the last day of the second data collection, and totalled 27 months. Compared to the total IQED population in 2011, these 3808 patients were more often patients with type 1 diabetes (35.3 % vs. 29.5 % of the total IQED population, *p* < 0.001). In general, the selected type 1 diabetes patients were younger (49 vs. 62 years old, *p* < 0.001) and had a longer diabetes duration (21 vs. 17 years old, *p* < 0.001), whereas the selected type 2 diabetes patients were older (70 vs. 62 years old, *p* < 0.001), had a slightly longer diabetes duration (18 vs. 17 years old, *p* < 0.01) and were more often female (52 vs. 49 %, *p* < 0.05) compared to the total IQED population.

Study cohorts were constructed to develop measures for the control of lipids (based on LDL cholesterol and non-HDL cholesterol), and blood pressure. Patients without, for their registration in 2011, an LDL cholesterol value (or a non-HDL cholesterol value for the measures based on non-HDL cholesterol, see below), a known status (presence or absence) of cardiovascular (CV) history (defined as a history of myocardial infarction (MI), cerebral vascular accident (CVA), percutaneous coronary intervention (PCI), coronary artery bypass graft (CABG) or transient ischemic attack (TIA)), or a known status (presence or absence) of end-stage kidney disease (defined as a history of kidney transplantation, peritoneal dialysis or haemodialysis) were excluded. Patients without a known lipid-lowering treatment status (treated or not treated, for both statins and fibrates) for their registration in 2009 and in 2011, were also excluded. This resulted in a final LDL cholesterol cohort of 1673 patients and a final non-HDL cholesterol cohort of 3421 patients. Excluded patients of both cohorts did not differ from the final sample in a clinically meaningful way in terms of age, sex, and diabetes duration.

The index value was defined as the most recent value within the measurement period, i.e. the value recorded for the data collection in 2011. The LDL cholesterol value was calculated by the Friedewald formula [12] for patients with a triglyceride value <400 mg/dl and for whom lipids were measured on a fasted blood sample. As the number of patients for whom an LDL cholesterol index value could be calculated strongly decreased when requiring a fasted sample and triglyceride value <400 mg/dl, we used non-HDL cholesterol as an alternative [13]. The non-HDL cholesterol value was calculated by subtracting the HDL cholesterol value from the total cholesterol value. The target values for non-HDL cholesterol were 30 mg/dl higher compared to the target values for LDL cholesterol.

Details about the construction of the cohort and development of the measures based on blood pressure can be found in Additional file 1.

### Measure development

**Fig. 1:**
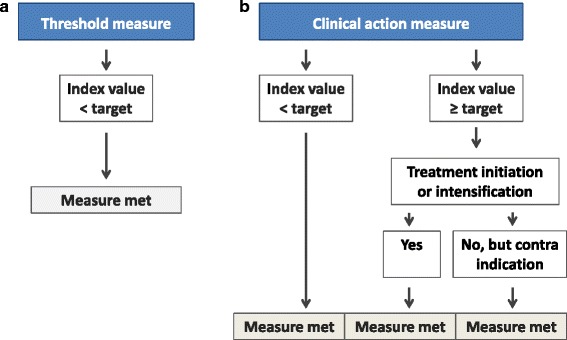
Flowchart describing the construction of the threshold measure and clinical action measure. Flowchart used to determine whether a patient met the threshold measure (Fig. 1a) or the clinical action measure (Fig. 1b)

We evaluated two quality measures: (1) the TM, and (2) the CAM. The criteria for meeting the TM and the CAM are shown in Fig. 1a and b, respectively. Creating the TM requires the presence of an LDL or non-HDL cholesterol index value, whereas the CAM requires, in addition, a known lipid-lowering treatment status and a known contraindication status. The LDL and non-HDL cholesterol index values were used as starting point.

### Threshold measure (TM)

Patients were defined as receiving good quality of care when a target value was reached. Based on the guidelines used at the time of the study [13–15], <100 mg/dl was used as target value for LDL cholesterol (<70 mg/dl for those with CV history), and <130 mg/dl for non-HDL cholesterol (<100 mg/dl for those with CV history).

### Clinical action measure (CAM)

Patients were defined as receiving good quality of care when the target value was reached or when an appropriate clinical action (treatment initiation or intensification) was taken between 2009 and 2011. If there was no treatment initiation or intensification recorded, patients also met the measure when a contraindication was recorded.

The same target values were used as for the TM. Treatment initiation or intensification was investigated by comparing the treatment regimen recorded in 2009 to the treatment regimen recorded in 2011. In the IQED questionnaire, centres could indicate whether the patient was treated (=1) or not (=0) with statins or fibrates, respectively. Patients were considered non-treated when treatment with neither statins nor fibrates was recorded (statins = 0 & fibrates = 0). As ezetimibe was only a part of the questionnaire from the data collection in 2011, this class of lipid-lowering drugs was not taken into account in this study.

As we did not have information on drug dose, treatment intensification was defined as the addition of an extra class of lipid-lowering drugs to the existing treatment regimen. A switch from one drug class to another was not considered as intensification, although it might be an appropriate action upon an indicated side effect, or lack of therapeutic effect.

Treatment initiation was defined as going from no treatment in 2009 to any treatment in 2011.

End-stage kidney disease, defined as the presence of kidney transplantation, peritoneal dialysis or haemodialysis, was used as a possible contraindication for treatment intensification or initiation.

### Statistical analysis

Patient characteristics were summarized as means (95 % confidence interval (CI)) or as frequencies (percentage). Missingness of data elements was investigated. The evolution of the proportion of patients treated was investigated between the 2009 and 2011 data collections. The total number of patients meeting the TM and the CAM were determined, as well as the number of patients per individual measure criterion. The centre-specific score for each measure was calculated. Funnel plots were used to detect statistical outliers: centres with a score of 2 (or 3) standard deviations (SD) above or below the overall score [16]. We categorized centres in three ranges: the normal range (within 2 SDs around the overall score), the high-outlier range (>2 SDs above the overall score) and the low-outlier range (>2 SDs below the overall score). Finally, characteristics of patients meeting the measure were compared with those of patients not meeting the measure. Most analyses were stratified by diabetes type. The *t*-test was used to compare continuous variables. The χ^2^-test was used to compare categorical variables. The Wilcoxon-test was used to compare the medians of the distributions of centre-specific indicator scores. Statistical significance was defined as *p*-value <0.05. All analyses were done in R, version 2.15.2.

## Results

### Study cohort and patient characteristics

Two consecutive data collections were used to construct a cohort followed in a measurement period from 2009 to 2011. Patient characteristics of the non-HDL cholesterol cohort at the end of the measurement period are summarized in Table 1. The characteristics of patients for whom an LDL cholesterol index value could be calculated did not significantly differ from patients for whom it could not.

**Table 1:** Patient characteristics

Measurement period	2009–2011			
Months	27			
Patients	3421			
Patient characteristics				
Diabetes type	Type 1		Type 2	
	(value)	(n)	(value)	(n)
Number of patients		1234		2187
Age (years), mean (95 % CI)	49 (48–50)		70 (69–70)	
Diabetes duration (years), mean (95 % CI)	21 (20–22)	−12	17 (17–18)	−111
Male, %	60		48	
Hba1c (%), mean (95 % CI)	7.9 (7.8–8.0)	−2	7.5 (7.4–7.5)	−13
Systolic blood pressure (mm Hg), mean (95 % CI)	128 (127–129)	−8	135 (135–136)	−20
Diastolic blood pressure (mm Hg), mean (95 % CI)	74 (74–75)	−8	74 (74–75)	−20
Total cholesterol (mg/dl), mean (95 % CI)	178.7 (176.8–180.6)		164.6 (163.1–166.1)	
non-HDL cholesterol (mg/dl), mean (95 % CI)	114.2 (112.5–116.0)		114.2 (112.8–115.6)	
LDL cholesterol (mg/dl), mean (95 % CI)	93.3 (91.7–94.8)	−666	84.1 (82.9–85.3)	−1082
CV-hist1, %	5.6		25.3	−4
CV-hist2, %	7.3		31.1	
CV-hist3, %	9.6	−113	36.6	−157
Microvascular complication, %	54.1	−224	74.9	−333

Patient characteristics at the end of the measurement period of the type 1 diabetes and type 2 diabetes patients from the non-HDL cholesterol cohort. Values are represented as means with their 95 % confidence interval (95 % CI), or as percentage (%). The negative values indicate the number of incomplete records that were not taken into account for the calculation. CV-hist1 is defined as a history of MI, CABG or PCI. CV-hist2 is defined as a history of MI, CABG, PCI, TIA or CVA. CV-hist3 is defined as a history of MI, CABG, PCI, TIA, CVA, peripheral bypass, or the absence of foot pulses. Microvascular complication is defined as any of the following: albuminuria ≥30 mg/dl, creatinine ≥1.5 mg/dl, dialysis or kidney transplantation, retinopathy, neuropathy, and foot ulcer or amputation

### Creating the measure: availability and evolution of required data elements

We first investigated the proportion of patients registered in both data collections for whom the separate required data elements were recorded (Additional file 2). An LDL cholesterol index value could be calculated for 70–75 % of the patients. This percentage was reduced to 44–47 % when taking into account the requirement of a fasted blood sample and a triglyceride value <400 mg/dl (data not shown). A non–HDL cholesterol index value could be calculated for 95–96 % of the patients. The lipid-lowering treatment status, contraindications status and CV history were completed for >95 % of the study population. Completeness of the required data elements of patients registered in both 2009 and 2011 did not differ from the completeness of the required data elements in the total IQED population.

Next, we investigated in detail the treatment status over time in our final study cohorts (Additional file 3). In general, the greatest proportion of patients treated with either statins or fibrates were patients with type 2 diabetes. The proportion of patients treated at the end of the measurement period was consistently and significantly higher compared to the start of the measurement period indicating that overall, additional patients started treatment within the given period. For the LDL cholesterol cohort, this percentage increased from 37.9 % type 1 diabetes patients in 2009 to 46.8 % in 2011 (*p* < 0.001) and from 74.5 % type 2 diabetes patients in 2009 to 80.3 % in 2011 (*p* < 0.001) in the LDL cholesterol cohort. These evolutions were similar in the non-HDL cholesterol cohort and confirmed in the total IQED population (data not shown).

Finally, we investigated treatment initiation and intensification at the individual patient level. In the LDL cholesterol cohort, 71 of the 568 type 1 diabetes patients (12.5 %) started treatment and two patients (0.3 %) intensified their treatment between 2009 and 2011. Of the 1105 type 2 diabetes patients, 109 patients (9.9 %) started treatment and 21 patients (1.9 %) intensified their treatment. These proportions were similar in the non-HDL cholesterol cohort.

### Meeting the measure: TM vs. CAM

The criteria for meeting the TM and CAM are shown in Fig. 1. Target values were adjusted for the presence or absence of CV history.

60.2 % of the type 1 diabetes patients and 63.0 % of the type 2 diabetes patients met the TM based on LDL cholesterol (Table 2). Patients without CV history were significantly more likely to meet their target value compared to those with CV history (62.8 % vs. 31.9 % of the type 1, *p* < 0.05, and 72.9 % vs. 39.5 % of the type 2 diabetes patients, *p* < 0.001). Looking at the TM based on non-HDL cholesterol, 70.5 % of the type 1 diabetes patients and 61.7 % of the type 2 diabetes patients met the TM. Again more patients without CV history met their target value compared to those with CV history (Table 2).

**Table 2:** Number and percentage of patients meeting the threshold measure and the clinical action measure

Diabetes type	Type 1		Type 2	
	(value)	(n)	(value)	(n)
Threshold measure				
Below target value				
LDL index value				
Total, patients (%)	342 (60.2)	568	696 (63.0)	1105
With CV history (LDL < 70 mg/dl), patients (%)	15 (31.9)	47	130 (39.5)	329
Without CV history (LDL < 100 mg/dl), patients (%)	327 (62.8)	521	566 (72.9)	776
non-HDL index value				
Total, patients (%)	870 (70.5)	1234	1349 (61.7)	2187
With CV history (non-HDL < 100 mg/dl), patients (%)	46 (51.1)	90	285 (41.9)	680
Without CV history (non-HDL < 130 mg/dl), patients (%)	824 (72.0)	1144	1064 (70.6)	1507
Clinical action measure				
Treatment and contraindication status				
LDL index value				
LDL ≥ 70 mg/dl, patients				
Treatment initiation or intens. [init.]	3 [3]		27 [23]	
Contraindication	2		4	
LDL ≥ 100 mg/dl, patients				
Treatment initiation or intens. [init.]	23 [22]		26 [23]	
Contraindication	2		5	
non-HDL index value				
non-HDL ≥ 100 mg/dl, patients				
Treatment initiation or intens. [init.]	3 [3]		51 [36]	
Contraindication	2		18	
non-HDL ≥ 130 mg/dl, patients				
Treatment initiation or intens. [init.]	42 [40]		53 [46]	
Contraindication	2		3	
Total				
LDL index value				
Total, patients (%)	372 (65.5) °		758 (68.6) °	
With CV history, patients (%)	20 (42.5) ^$^		161 (48.9) °	
Without CV history, patients (%)	352 (67.6) °		597 (76.9) °	
non-HDL index value				
Total, patients (%)	919 (74.5) °		1474 (67.4) °	
With CV history, patients (%)	51 (56.7) °		354 (52.1) °	
Without CV history, patients (%)	868 (75.9) °		1120 (74.3) °	

The denominator is represented by (n). The target of the index value for patients with cardiovascular history (defined as the presence of a MI, CABG, PCI, TIA or CVA) is lower than for those without cardiovascular history. Data are represented for the total number of type 1 or type 2 diabetes patients, as well as in the absence or presence of cardiovascular history. The proportion with an index value equal to or above the threshold, but with a recorded treatment initiation or intensification, or with a recorded contraindication for treatment intensification or initiation, is represented as number of patients. Statistical significance between the percentage of patients meeting the threshold measure and the clinical action measure was tested by the χ^2^-test: *p* < 0.001 (°), *p* < 0.01 (^$^) and *p* < 0.05 (*) vs. threshold measure

Meeting the CAM is the result of an index value below target, or an index value above target but with an indicated treatment initiation or intensification. Table 2 shows that among type 1 diabetes patients with an LDL cholesterol index value above target, three patients with and 23 without CV history had treatment initiation or intensification during the measurement period. In addition, four patients had a reported contraindication to treatment initiation or intensification. As a result, the proportion of patients meeting the measure significantly increased from 60.2 % based on the TM to 65.5 % based on the CAM (*p* < 0.001). Similarly, among type 2 diabetes patients with an LDL cholesterol index value above target, 27 patients with and 26 without CV history had treatment initiation or intensification during the measurement period. In addition, nine patients had a reported contraindication to treatment initiation or intensification. This lead to a significant increase in the proportion of patients meeting the measure: from 63.0 % based on the TM to 68.6 % based on the CAM (*p* < 0.001).

A comparable increase was found for the measures based on non-HDL cholesterol (Table 2).

Interestingly, the increase in proportion meeting the measure from TM to CAM was higher among patients with CV history (6–11 %) than among those without (4–5 %).

### Provider profiling according to the TM and the CAM

Table 3 summarizes the centre specific scores for the two measures. Only centres with five or more patients in the cohort were included. This led to a reduction from 95 centres that participated in IQED and had patients registered in both 2009 and 2011, to 63 and 89 centres for the measures based on LDL cholesterol and non-HDL cholesterol respectively. The overall scores, using the total number of patients as a denominator after excluding these centres, were not significantly different from those in Table 2 (data not shown).

**Table 3:** Overall mean percentage meeting the measure and centre specific percentiles

	Overall	P10	P50	P90
Threshold measure				
LDL index value				
Number of centres	63			
(Number of patients per centre, range)	(5–99)			
Total number of patients	1635			
Total score (%)	61.7	48.4	63.6	76.4
non-HDL index				
Number of centres	89			
(Number of patients per centre, range)	(5–126)			
Total number of patients	3411			
Total score (%)	64.8	49.6	64.7	77.1
Clinical action measure				
LDL index value				
Number of centres	63			
(Number of patients per centre, range)	(5–99)			
Total number of patients	1635			
Total score (%)	67.0	54.7	68.8 ^$^	81.0
non-HDL index value				
Number of centres	89			
(Number of patients per centre, range)	(5–126)			
Total number of patients	3411			
Total score (%)	70.0	55.7	69.2 °	80.6

Overall mean percentage meeting the measure for each measure, and the centre specific 10th, 50th and 90th percentiles. Only centres with five or more patients were taken into account. Statistical significance between median centre specific score for the threshold measure and the clinical action measure was tested by the Wilcoxon-test: *p* < 0.001 (°) and *p* < 0.01 (^$^) vs. the threshold measure

The median centre-specific score for the CAM was significantly higher than for the TM: 68.8 % (CAM) vs. 63.6 % (TM) for the LDL cholesterol measures (*p* < 0.01), and 69.2 % (CAM) vs. 64.7 % (TM) for the non-HDL cholesterol measures (*p* < 0.001). Between-centre variability, as demonstrated by the range between the 10th and 90th percentile, was comparable for both the TM and the CAM (Table 3).

**Fig. 2:**
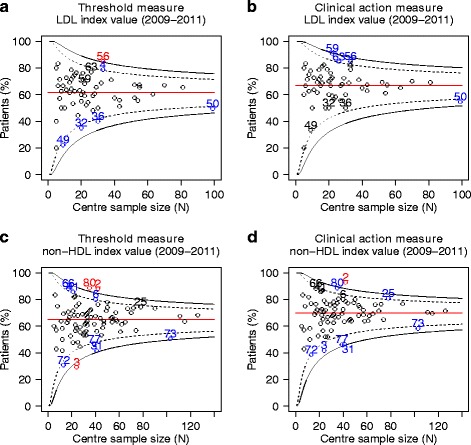
Funnel plots-control of lipids. Funnel plots of the centre specific scores for the threshold measure and the clinical action measure for the control of lipids. Each circle represents a centre. Centres were given a random identifier. Only centres with five or more diabetes patients were included. The red line represents the overall mean proportion of patients meeting the measure. Centres with a score more than two standard deviations from the overall mean proportion (black dashed line) are marked in blue and centres with a score more than three standard deviations from the overall mean proportion (black solid line) are marked in red

Funnel plots were created to detect statistical outliers (Fig. 2a-d). The funnel plot for the TM based on LDL cholesterol shows two high outliers (centres 56 and 4), and four low outliers (centres 49, 32, 36 and 50) (Fig. 2a). The remaining 57 centres (90 %) were in the normal range. The funnel plot for the CAM based on LDL cholesterol shows that the overall score and the centre-specific scores increased compared to the TM (Fig. 2b). Three of the four original low outliers (centres 49, 32 and 36) moved to the normal range using the CAM. One original high outlier (centre 4) moved to the normal range. In addition, two new centres (centres 63 and 59) moved to the high-outlier range. In total, 59 centres (94 %) were within the normal range using the CAM.

The funnel plot for the TM based on non-HDL cholesterol shows five high outliers (centres 66, 41, 6, 80 and 2), and five low outliers (centres 72, 3, 77, 31 and 73) (Fig. 2c). The remaining 79 centres (89 %) were in the normal range. Again, the funnel plot for the CAM based on non-HDL cholesterol shows that the overall score and the centre-specific scores increased compared to the TM (Fig. 2d). All original low outliers remained identified as low outlier. Three original high outliers (centres 66, 41 and 6) moved to the normal range. One new centre (centre 25) moved from the normal range to the high-outlier range. In total, 81 centres (91 %) were within the normal range using the CAM.

### Provider profiling applied to the control of blood pressure

**Fig. 3:**
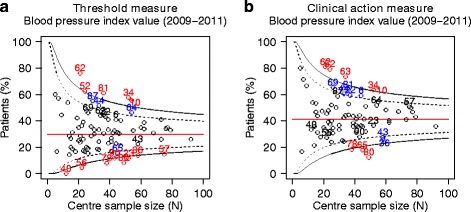
Funnel plots-control of blood pressure. Funnel plots of the centre specific scores for the threshold measure and the clinical action measure for the control of blood pressure. Each circle represents a centre. Centres were given an anonymous rank number. Only centres with five or more diabetes patients were included. The red line represents the overall mean proportion of patients meeting the measure. Centres with a score more than two standard deviations from the overall mean proportion (black dashed line) are marked in blue, centres with a score more than three standard deviations from the overall mean proportion (black solid line) are marked in red

We applied the same principle to the control of blood pressure, and investigated provider profiling. For these analyses, only centres with five or more patients in the cohort were included, leading to a total of 3609 patients treated over 90 centres. Again the median centre-specific score for the CAM was significantly higher than for the TM: 40.7 % (CAM) vs. 30.2 % (TM, *p* < 0.001). The funnel plots for the TM shows eight high outliers (centres 62, 52, 87, 44, 81, 34, 10 and 64) and eight low outliers (centres 40, 56, 78, 90, 80, 23, 36 and 57) (Fig. 3a). The remaining 74 centres (82 %) were in the normal range. The funnel plot for the CAM shows that two original high outliers (centres 87 and 64) and five original low outliers (centres 40, 56, 90, 23 and 57) moved to the normal range (Fig. 3b). In addition, four new centres (centres 69, 63, 22 and 6) moved to the high-outlier range, and one centre (centre 43) moved to the low-outlier range. In total, 75 centres (83 %) were within normal range using the CAM.

### Characteristics of patients meeting the measure and of those not meeting the measure

Table 4 shows the patient characteristics of those not meeting a measure (TM-CAM-), not meeting the TM but meeting the CAM (TM-CAM+), and meeting the TM and CAM (TM + CAM+) based on LDL cholesterol (Table 4a) and on non-HDL cholesterol (Table 4b).

**Table 4:** Patient characteristics associated with meeting the TM and the CAM

Diabetes type	T1			T2		
Meeting the measure	TM–CAM−	TM–CAM +	TM + CAM +	TM–CAM−	TM-CAM +	TM + CAM +
A.	LDL cholesterol					
n	196	30	342	347	62	696
Age (years), mean	48	51	51 *	69	71	69
Diabetes duration (years), mean	20	20	22	17	17	18
Time before start insulin therapy (years), mean	0.2	0.3	0.5	7.8	8.2	8.6
Duration insulin therapy (years), mean	20	20	21	9.2	8.4	9.2
Smoking status (%)						
Never smoked	63.7	50.0	65.8	65.6	59.3	66.9
Ex-smoker	13.5	23.3	12.7	25.0	28.8	24.2
Smoker	22.8	26.7	21.5	9.4	11.9	8.9
Presence of complication (%)						
CV_hist1 (° for T2D)	8.7	10.0	4.1	40.9	38.7	15.5
CV_hist2 (° for T1D, ° for T2D)	13.8	16.7	4.4	48.4	50.0	18.7
CV_hist3 (^$^ for T1D, ° for T2D)	15.4	18.5	6.2	52.4	55.2	24.2
Microvascular complication	51.6	60.9	53.8	71.7	81.8	73.2
Absent foot pulses or abnormal sensitivity	14.2	16.0	16.0	36.3	43.1	33.5
Treated with (%)						
Antiplatelet drugs (* for T1D)	29.1	50.0	36.9	71.6	69.4	74.5
Lipid-lowering drugs (^$^ for T1D, ^$^ for T2D)	32.6	93.3	50.9	71.5	93.5	83.5
Antihypertensive drugs	40.8	46.7	51.0	86.4	87.1	87.0
Oral antidiabetic drugs (° for T2D)	7.7	13.3	8.9	51.3	33.9	58.4
B.	non-HDL cholesterol					
n	315	49	870	713	125	1349
Age (years), mean	47	49	50 ^$^	69	70	70
Diabetes duration (years), mean	19	21	22 ^$^	17	17	18
Time before start insulin therapy (years), mean	0.2	0.4	0.3	7.9	7.5	8.0
Duration insulin therapy (years), mean	18	21	22 °	8.9	8.7	9.5
Smoking status (%)						
Never smoked	57.2	61.7	65.0	60.5	60.4	65.0
Ex-smoker	15.1	19.1	14.9	27.8	29.7	25.2
Smoker	27.6	19.1	20.0	11.7	9.9	9.8
Presence of complication (%)						
CV_hist1 (° for T1D, ° for T2D)	9.5	8.2	4.0	37.5	40.3	17.4
CV_hist2 (° for T1D, ° for T2D)	12.4	10.2	5.3	45.7	55.2	21.1
CV_hist3 (^$^ for T1D, ° for T2D)	13.9	14.6	7.8	50.1	60.3	27.0
Microvascular complication (* for T2D)	52.1	73.0	53.8	74.7	85.3	73.9
Absent foot pulses or abnormal sensitivity (* for T2D)	12.0	15.4	14.8	37.0	43.3	32.4
Treated with (%)						
Antiplatelet drugs (^$^ for T1D)	22.2	36.7	33.0	72.1	69.6	72.4
Lipid-lowering drugs (° for T1D, ° for T2D)	29.5	98.0	45.2	69.1	95.2	81.9
Antihypertensive drugs (^$^ for T1D)	35.9	44.9	45.8	84.9	83.9	86.2
Oral antidiabetic drugs (^$^ for T2D)	8.0	8.2	8.4	53.7	41.9	56.4

Patient characteristics that were associated with meeting the TM and the CAM based on LDL cholesterol (Table 4a) and non-HDL cholesterol (Table 4b). Mean age, diabetes duration, time before start insulin therapy and duration of insulin therapy is compared between the population neither meeting the TM nor the CAM (TM-CAM-), those not meeting the TM but meeting the CAM (TM-CAM+) and those meeting the TM and the CAM (TM + CAM+). Statistical significance between means was tested with the Pairwise Wilcox test, Holm adjustment: *p* < 0.001(°), *p* < 0.01 (^$^) and *p* < 0.05 (*) vs. not meeting the TM or the CAM (TM-CAM-). The proportion of patients with a complication, treated with drugs, and the distribution of smoking status is compared between the population neither meeting the TM nor the CAM (TM-CAM-), those not meeting the TM but meeting the CAM (TM-CAM+) and those meeting the TM and the CAM (TM + CAM+). Statistical significance between percentages was tested with the χ^2^-test: *p* < 0.001 (°), *p* < 0.01 (^$^) and *p* < 0.05 (*)

Based on the measures with LDL cholesterol, diabetes type was not associated with meeting the TM or CAM (TM-CAM-: 34.5 % type 1 vs. 31.4 % type 2 diabetes patients, TM-CAM+: 5.3 % type 1 vs. 5.6 % type 2 diabetes patients, TM + CAM+: 60.2 % type 1 vs. 63.0 % type 2 diabetes patients). Based on the measures with non-HDL cholesterol, type 1 diabetes patients were more likely to meet the TM or CAM than patients with type 2 diabetes (TM-CAM-: 25.5 % type 1 vs. 32.6 % type 2 diabetes patients, TM-CAM+: 4.0 % type 1 vs. 5.7 % type 2 diabetes patients, TM + CAM+: 70.5 % type 1 vs. 61.7 % type 2 diabetes patients, χ^2^-test: *p* < 0.01).

In general, type 1 diabetes patients meeting the TM and CAM were older, had a longer diabetes duration and a slightly longer duration of insulin therapy than those neither meeting the TM nor the CAM. These associations with age and diabetes duration were less pronounced for the type 2 diabetes patients. Patients meeting the TM and the CAM had less frequently CV history than those neither meeting the TM nor the CAM, and the type 1 diabetic patients meeting the TM and the CAM were slightly more often treated.

Patients that did not meet the TM, but met the CAM had a slightly higher prevalence of microvascular complications and absence of foot pulses or abnormal sensitivity than those neither meeting the TM nor the CAM, and those meeting the TM and the CAM. They were in general more often treated, especially with lipid-lowering drugs.

## Discussion

This study shows that scoring quality of diabetes care within a centre based on a classical TM may be misleading, and that low-complexity data elements available from a nationwide quality improvement initiative can be used to construct a more fair measure of quality of care.

Our first objective was to investigate the feasibility of constructing a CAM for the control of lipids from an existing cross-sectional study including mostly low-complexity data elements. Although a major barrier to create such a more complex measure is the availability of the additional data elements [6], we showed that all additional data elements were present for >95 % of our study population, making the CAM a feasible measure of quality of care without affecting the number of patients on which quality of care is calculated. As the CAM requires longitudinal data, we combined two consecutive data collections, which allowed us to create a study population of which quality of care could be evaluated over time. We did not have information on the writing of prescriptions or refills, or on drug dosages. As a result, we were not able to give credit to patients that were already on maximum treatment dose, or had intensified their treatment by increasing the dose without adding a new class of drugs. However, information on drug dosages is a high-complexity data element, and in practice, few care providers are able to provide these data without a high additional burden. Currently, efforts promoting the use of electronic medical records containing extractable data fields and comprehensive automated data sources will decrease this burden. In turn, the CAM can be fine-tuned further for provider’s actions upon poor outcome.

Our second aim was to investigate how quality of care defined by the CAM related to that defined by the TM. In general, the use of the CAM will always lead to equal or higher scores of quality of care. Whereas the TM only gives credit to patients with an LDL (or non-HDL) cholesterol value below target, the CAM will in addition give credit to patients with an LDL (or non-HDL) cholesterol value above target when lipid-lowering treatment was initiated or intensified while respecting possible contraindications for treatment. We used end-stage kidney failure (transplantation, peritoneal dialysis or haemodialysis) as a possible contraindication, although the usefulness of statins in end-stage kidney failure is still debated in the literature [17]. Our results confirmed a systematic and significant increase in the score of quality of care based on CAM compared to TM. This increase was more pronounced when the target value was lower.

Meeting the measure of good care was associated with a number of patient characteristics. In general, patients meeting the TM (and consequently the CAM) were more often patients without CV history. Within the population of patients not meeting the TM, those meeting the CAM had a slightly higher prevalence of microvascular and foot complications compared to those not meeting the CAM. As treatment is a component of the CAM, they were more often treated, especially with lipid-lowering drugs.

We also found a general positive evolution in the proportion of patients treated during the measurement period. Some of these patients that started treatment will meet the TM as they had, at the time of the index value, an LDL (or non-HDL) cholesterol value below target. Nevertheless, our study showed that 30–40 % of these patients still remained to be picked up by the CAM, strengthening the use of the CAM over the TM. Indeed, there is a move away from one–time point, one-target-for-all, measures towards measures based on control of risk factors that take into account patient characteristics and the longitudinal nature of chronic care [2, 6, 10]. The classical TM only gives credit to a good intermediate outcome, favouring centres with less severe patients or patients that respond well to therapy. Poor outcome could reflect inadequate attention from physicians, but also individual patient factors [18]. In addition, one can speculate that if the quality score is used for public accountability or reimbursement, the temptation to deselect patients that are more difficult to control might exist. A more fair way of assessing quality of care also gives credit to an appropriate action upon a poor intermediate outcome.

Finally, we wanted to compare the reliability of provider profiling using the CAM and the TM. Using the CAM still leaves sufficient variance between centre-specific scores and room for further improvement, allowing it to be useful for benchmarking. The funnel plots showed that centres identified as performing better (or worse) than the others based on the TM were not by definition those that performed better (or worse) based on the CAM. This indicates that quality of care defined by the classical TM was overestimated (or underestimated). Original high outliers moving into the normal range were centres whose scores were overestimated using the TM. Indeed, for these centres, the CAM-induced increase in the centre-specific score was lower than the CAM-induced increase in the overall score. In other words, they lost relatively more points using the CAM compared to the other centres. The opposite is true for the original low outliers moving to the normal range, and for the centres moving from the normal range to the high-outlier range. These were centres whose scores were underestimated using the TM. For these centres, the CAM-induced increase in the centre-specific score was higher than the CAM-induced increase in the overall score. They gained relatively more points using the CAM compared to the other centres.

The principle of scoring quality of care based on a CAM is general and can be applied to the control of other intermediate outcomes [7, 19, 20]. Indeed, we applied the principle to the control of blood pressure. Similarly as for the control of lipids, the position related to the other centres changed for 13 % of the centres, indicating that judging quality of care based on a TM might be misleading.

The approach of our study makes it difficult to compare with other studies that created a CAM for the control of lipids in diabetes patients [7, 21, 22]. Whereas the other studies investigated the clinical action within a certain timeframe after a high-index LDL value, we studied the clinical actions taken before or at the moment of the high-index LDL value. IQED is a quality improvement initiative that investigates the care given to patients in the past year by asking the physicians to register the patient’s most recent values. Based on those values, centres are scored and ranked. We believe that credit should be given when a patient’s therapy has been initiated (or intensified) although the patient did not meet the target value (yet). Furthermore, we adjusted our target values according to CV history, moving away from the one-target-for-all measures.

In addition to the limitations already mentioned, our study has one additional limitation. Our measurement period was constructed by the combination of two consecutive cross-sectional data collections, and exceeded the 120 days mentioned by Sidorenkov et al. as the period in which clinical actions should be taken [10] and those used in other studies [7, 21, 22]. As a result, we might have missed intermediate test results and therapy changes. However, the diabetes stage in our study population was advanced: type 1 and type 2 diabetes patients on at least two insulin injections a day, and a high prevalence of micro- and macrovascular complications. One can speculate that our study population was less exposed to drastic changes in therapy and subsequent outcomes, and, in the context of more stable chronic care delivery, a longer measurement period may actually better fit our needs. In addition, our results showed that still 30 to 40 % of the patients that started therapy during the measurement period did not meet their target value, and consequently were reclassified from receiving suboptimal to good care using the CAM.

Nevertheless, as mentioned earlier, efforts should be made to further develop structured electronic medical records. The availability of extractable data fields and more automated data collection tools will decrease the burden to collect more high-complexity data elements reflecting clinician’s actions (such as drug dosages and changes in regimen), and allow to collect multiple recent values of a measurement (e.g. LDL cholesterol) within one data collection. The latter would provide a clearer picture of the evolution of a patient in relation to clinical actions.

## Conclusions

In summary, we created a more fair measure of quality of care that is immediately applicable for benchmarking feedback in a quality improvement initiative. Further development of the electronic health records will support optimization of the CAM.

## Additional files

Additional file 1: Cohort construction and measure development for the control of blood pressure. (DOCX 13 kb) Additional file 2: Missingness of required data elements used to build the threshold and clinical action measure. (DOCX 17 kb) Additional file 3: Evolution lipid-lowering treatment. (DOCX 14 kb)

## Abbreviations

CABG: Coronary artery bypass graft
CAM: Clinical action measure
CI: Confidence interval
CV history: Cardiovascular history
CVA: Cerebral vascular accident
HbA1c: Glycated haemoglobin
IQED: Initiative for quality improvement and epidemiology in patients with diabetes
LDL: Low-density lipoprotein
MI: Myocardial infarction
non-HDL: Non-high-density lipoprotein
PCI: Percutaneous coronary intervention
SD: Standard deviation
TIA: Transient ischemic attack
TM: Threshold measure
