# Supplementary material for: Clinical action measures improve the reliability of feedback on quality of care in diabetes centres: a retrospective cohort study
Source: BMC Health Serv Res. 2016 Aug 23;16(1):424. doi: 10.1186/s12913-016-1670-5 (PMC4995611; doi:10.1186/s12913-016-1670-5)
Supplement: Additional file 1: — Cohort construction and measure development for the control of blood pressure. (DOCX 13 kb) [file 12913_2016_1670_MOESM1_ESM.docx]

Additional file 1: Cohort construction and measure development for the control of blood pressure.

Patients without a blood pressure (BP) value registered in 2011, or without a known blood pressure-lowering treatment status (treated or not treated, for both angiotensin converting enzyme inhibitors (ACE-I) or angiotensin receptor blockers (ARB’s) and other antihypertensive drugs) for their registration in 2009 and in 2011, were also excluded. This resulted in a final BP cohort of 3616 patients.

Based on the TM, patients received good quality of care when their blood pressure was <130/80 mmHg. Based on the CAM, patients received good quality of care when their blood pressure was <130/80 mmHg, or when their blood pressure was ≥130/80 mmHg but a treatment initiation or intensification was indicated. Treatment initiation or intensification was investigated by comparing the treatment regimen recorded in 2009 to the treatment regimen recorded in 2011. In the IQED questionnaire, centres could indicate whether the patient was treated (=1) or not (=0) with respectively angiotensin converting enzyme inhibitors (ACE-I) and angiotensin receptor blockers (ARB’s), or other antihypertensive drugs. Patients were considered non-treated when treatment with neither ACE-I nor ARB’s nor other antihypertensive drugs was recorded (ACE-I/ARB = 0 & other = 0). Treatment initiation was defined as going from no treatment in 2009 to any treatment in 2011. We did not define a possible contraindication for treatment intensification or initiation.
