# Supplementary material for: Clinical action measures improve the reliability of feedback on quality of care in diabetes centres: a retrospective cohort study
Source: BMC Health Serv Res. 2016 Aug 23;16(1):424. doi: 10.1186/s12913-016-1670-5 (PMC4995611; doi:10.1186/s12913-016-1670-5)
Supplement: Additional file 2: — Missingness of required data elements used to build the threshold and clinical action measure. (DOCX 17 kb) [file 12913_2016_1670_MOESM2_ESM.docx]

Additional file 2: Missingness of required data elements used to build the threshold and clinical action measure.

Data elements from the IQED questionnaire were used to build the threshold and clinical action measure. The completeness of the required elements is compared between the total IQED population and the population of patients registered in both 2009 and 2011, and represented in the table below.

|  | **IQED population**  (cross sectional) | | | | **Study population**  (measurement period) | |
| --- | --- | --- | --- | --- | --- | --- |
|  | **2009** | | **2011** | | **2009 - 2011** | |
|  | **T1D** | **T2D** | **T1D** | **T2D** | **T1D** | **T2D** |
| Number of patients | 3437 | 7922 | 3530 | 8443 | 1344 | 2464 |
| LDL index, % missing set ^a^ | 18.3 | 20.9 | 23.0 | 26.8 | 25.1 | 29.6 |
| non-HDL index, % missing set  ^a^ | 5.9 | 6.4 | 3.9 | 5.9 | 3.6 | 5.0 |
| Statins, % missing ^b^ | 1.2 | 2.1 | 2.3 | 2.0 | 0.7  1.6 | 0.9  1.7 |
| Fibrates, % missing ^b^ | 1.5 | 2.4 | 2.8 | 3.0 | 0.5  1.7 | 1.5  2.6 |
| Contra indication, % missing ^c^ | 4.2 | 4.9 | 3.3 | 6.0 | 2.4 | 3.6 |
| CVhist2, % missing ^c^ | 2.1 | 3.2 | 1.6 | 3.1 | 1.3 | 2.2 |
| CHOL index, % missing | 3.9 | 4.0 | 2.7 | 3.8 | 2.5 | 3.1 |
| TG index, % missing | 5.8 | 5.4 | 3.4 | 5.0 | 3.3 | 4.4 |
| HDL index, % missing | 5.8 | 6.4 | 3.9 | 5.8 | 3.6 | 4.9 |
| Fasting status of index values, % missing | 17.0 | 19.3 | 22.0 | 25.1 | 24.7 | 28.3 |
| Centre specific IQR of fasting status of index values, % missing  (number of centres) | 3 -  23  (113) | 4 -  20  (112) | 3 -  25  (115) | 6 -  33  (114) | 0 -  33  (94) | 2 -  42  (91) |

^a^ For the LDL and non-HDL index values, the percentage of patients with an incomplete set of parameters necessary to calculate the index value is given. For the LDL index, the necessary parameters are a total cholesterol value, a HDL cholesterol value, a triglyceride value (TG) and an indication of the fasted status (fasted or not). The necessary parameters for the non-HDL index are a total cholesterol value and a HDL cholesterol value.

^b^ For the study population, the percentage missing data on statin and fibrate treatment is represented as the % missing data on treatment (treated or not treated) at start of the measurement period (2009) and the % missing data at the end of the measurement period (2011).

^c^ A status (presence or absence) of contra indication (history of kidney transplantation and/or peritoneal dialysis or haemodialysis) or cardiovascular history (history of a MI, CABG, PCI, TIA or CVA) was considered as known for the study population when this was indicated at the end of the measurement period (2011).
