# Supplementary material for: Clinical action measures improve the reliability of feedback on quality of care in diabetes centres: a retrospective cohort study
Source: BMC Health Serv Res. 2016 Aug 23;16(1):424. doi: 10.1186/s12913-016-1670-5 (PMC4995611; doi:10.1186/s12913-016-1670-5)
Supplement: Additional file 3: — Evolution lipid-lowering treatment. (DOCX 14 kb) [file 12913_2016_1670_MOESM3_ESM.docx]

Additional file 3: Evolution lipid-lowering treatment.

The table below shows the percentage of type 1 and type 2 diabetes patients in our study population treated with hypolipidemic drugs (statins or fibrates). Statistical significance between the proportion of patients treated at the start of the measurement period to the proportion of patients treated at the end of the measurement was tested with the Pearson’s Chi-squared Test for count data: p<0.001 (°), p<0.01 (^$^) and p<0.05 (*) vs. start of the measurement period.

|  | **2009-2011** | |
| --- | --- | --- |
|  | **Type 1** | **Type 2** |
| **LDL cohort** |  |  |
| Number of patients | 568 | 1105 |
| lipid-lowering treatment |  |  |
| at start of measurement period, % | 37.9 | 74.5 |
| at end of measurement period, % | 46.8 ° | 80.3 ° |
| **non-HDL cohort** |  |  |
| Number of patients | 1234 | 2187 |
| lipid-lowering treatment |  |  |
| at start of measurement period, % | 34.5 | 73.4 |
| at end of measurement period, % | 43.3 ° | 78.5 ° |
